# 714. Genotypic Resistance in *Acinetobacter* as a Predictor for Patient Outcomes

**DOI:** 10.1093/ofid/ofad500.776

**Published:** 2023-11-27

**Authors:** Chetan Jinadatha, Sorabh Dhar, John David Coppin, Thanuri Navarathna, Keith S Kaye, Hosoon Choi, Piyali Chatterjee

**Affiliations:** Central Texas Veterans Health Care System, Temple, Texas; Wayne State University/Detroit Medical Center, John Dingell VAMC, Detroit, Michigan; Central Texas Veterans Health Care System, Temple, Texas; Central Texas Veterans Health Care System, Temple, Texas; Rutgers Robert Wood Johnson Medical School; Central Texas Veterans Health Care System, Temple, Texas; Central Texas Veterans Health Care System, Temple, Texas

## Abstract

**Background:**

Infections caused by *Acinetobacter baumannii* are frequently difficult to treat with currently available antibiotics. Oxacillinases (blaOXA-23), beta-lactamase (blaTEM-1D) are known to contribute to antibiotic resistance. The purpose of this study was to identify if resistance genes such as blaOXA-23 and/or blaTEM-1D contribute to adverse patient outcomes such as prolonged infection leading to increased duration of stay and mortality in hospitalized patients.

**Methods:**

Clinical patient isolates were collected from two acute care hospitals, a 383-bed, and a 248-bed hospital in the Detroit metropolitan area. A total of 115 isolates samples and with complete clinical data sets were collected from 2 medical ICUs, 2 surgical ICU wards and 12 non-ICU surgical wards between 2017-2021. Resistance genes were analyzed using Resfinder (version 4.0) following whole genome sequencing (Illumina, NextSeq). Separate Bayesian regression models were used for the outcomes of interest: length of stay (LOS) and 30-day mortality, for each gene, blaOXA-23 and blaTEM-1D, as a predictor and adjusting for age and sex, using negative binomial models for LOS and logistic regression for mortality. Full models were fit to each outcome that included the interaction of both genes and adjusted for age, gender, hospital unit, respiratory infection, diabetes, COPD, hypertension, and renal dysfunction.

**Results:**

The patient clinical characteristics between the resistant and susceptible *Acinetobacter* groups were matched (Table1). There was little evidence for the genes affecting either 30-day mortality or LOS for the full models. There was some evidence for increased odds of mortality with the presence of blaOXA-23, 0.75 (95% uncertainty interval: -0.06, 1.60), or both blaOXA-23 and blaTEM-1D, 0.85 (-0.2, 2.0) but not at the 95% level (Figure 1, Table 2). There was little evidence for an effect for the genes on LOS.

**Figure:**
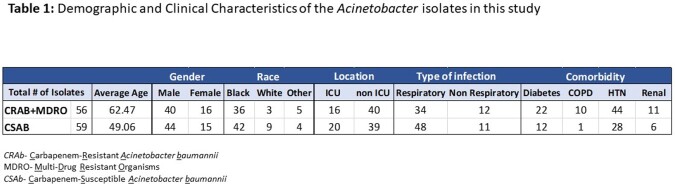

**Figure:**
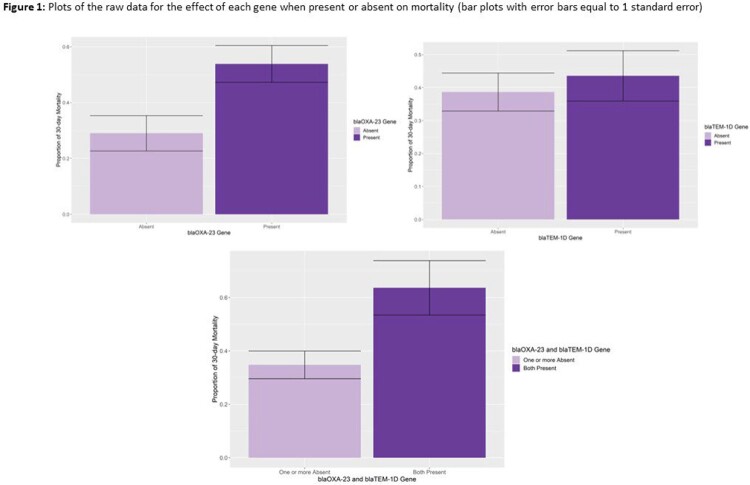

**Figure:**
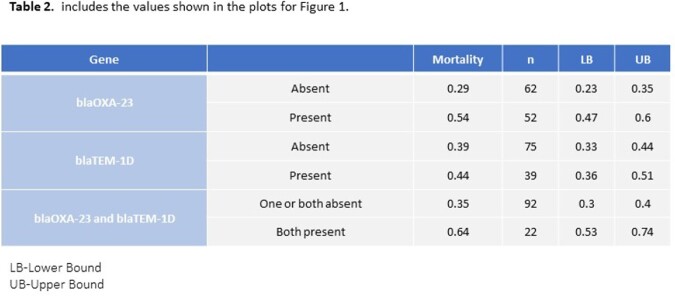

**Conclusion:**

Although there was a trend towards resistance genes affecting outcomes, the full analysis indicated no relationship between resistance genes and overall outcomes. Other factors such as advanced age, and comorbidities may be contributing to the mortality. In the future, factors that contribute to adverse clinical outcomes using a larger cohort will be explored.

**Disclosures:**

**Keith S. Kaye, MD, MPH**, Abbvie: Advisor/Consultant|Abbvie: Honoraria|Entasis: Advisor/Consultant|Entasis: Honoraria|GSK: Advisor/Consultant|GSK: Honoraria|Merck: Advisor/Consultant|Merck: Honoraria|Shionogi: Advisor/Consultant|Shionogi: Honoraria|VenatoRx: Advisor/Consultant|VenatoRx: Honoraria

